# Supplementary material for: Transcriptome Analysis Reveals Vimentin-Induced Disruption of Cell–Cell Associations Augments Breast Cancer Cell Migration
Source: Cells. 2022 Dec 13;11(24):4035. doi: 10.3390/cells11244035 (PMC9776984; doi:10.3390/cells11244035)
Supplement: Supplementary file 1 [file cells-11-04035-s001.zip › DEGs and enrichment analysis.zip!/DEGs and enrichment analysis/WT_VIMvsVector KEGG/UP/WT_VIMvsVector_UP.pathway_map.zip!/WT_VIMvsVector_UP.pathway_map/pathway/WT_VIMvsVector.html]

WT\_VIMvsVector.utf8.md


###WT\_VIMvsVector KEGG通路富集列表 \*\*\*

| KEGGID | Description | GeneRatio | BgRatio | pvalue | padj | geneID | geneName | keggID | Count |
| --- | --- | --- | --- | --- | --- | --- | --- | --- | --- |
| hsa03030 | DNA replication | 9\|135 | 41\|9228 | 0.0000000 | 0.0000011 | ENSG00000073111/ENSG00000100297/ENSG00000166508/ENSG00000168496/ENSG00000049541/ENSG00000062822/ENSG00000106628/ENSG00000014138/ENSG00000198056 | MCM2/MCM5/MCM7/FEN1/RFC2/POLD1/POLD2/POLA2/PRIM1 | hsa:4171/hsa:4174/hsa:4176/hsa:2237/hsa:5982/hsa:5424/hsa:5425/hsa:23649/hsa:5557 | 9 |
| hsa04110 | Cell cycle | 15\|135 | 163\|9228 | 0.0000000 | 0.0000013 | novel.507/ENSG00000093009/ENSG00000127564/ENSG00000166851/ENSG00000117399/ENSG00000073111/ENSG00000085840/ENSG00000135476/ENSG00000100297/ENSG00000166508/ENSG00000101412/ENSG00000145386/ENSG00000164045/ENSG00000157456/ENSG00000116670 | -/CDC45/PKMYT1/PLK1/CDC20/MCM2/ORC1/ESPL1/MCM5/MCM7/E2F1/CCNA2/CDC25A/CCNB2/MAD2L2 | hsa:1870/hsa:8318/hsa:9088/hsa:5347/hsa:991/hsa:4171/hsa:4998/hsa:9700/hsa:4174/hsa:4176/hsa:1869/hsa:890/hsa:993/hsa:9133/hsa:10459 | 15 |
| hsa04914 | Progesterone-mediated oocyte maturation | 8\|135 | 120\|9228 | 0.0003622 | 0.0247493 | ENSG00000082175/ENSG00000127564/ENSG00000166851/ENSG00000145386/ENSG00000164045/ENSG00000164742/ENSG00000157456/ENSG00000116670 | PGR/PKMYT1/PLK1/CCNA2/CDC25A/ADCY1/CCNB2/MAD2L2 | hsa:5241/hsa:9088/hsa:5347/hsa:890/hsa:993/hsa:107/hsa:9133/hsa:10459 | 8 |
| hsa04114 | Oocyte meiosis | 8\|135 | 171\|9228 | 0.0035577 | 0.1823322 | ENSG00000082175/ENSG00000127564/ENSG00000166851/ENSG00000117399/ENSG00000135476/ENSG00000164742/ENSG00000157456/ENSG00000116670 | PGR/PKMYT1/PLK1/CDC20/ESPL1/ADCY1/CCNB2/MAD2L2 | hsa:5241/hsa:9088/hsa:5347/hsa:991/hsa:9700/hsa:107/hsa:9133/hsa:10459 | 8 |
| hsa05166 | Human T-cell leukemia virus 1 infection | 10\|135 | 261\|9228 | 0.0048889 | 0.1884125 | novel.507/ENSG00000117399/ENSG00000135476/ENSG00000101412/ENSG00000145386/ENSG00000164742/ENSG00000117394/novel.600/ENSG00000164362/ENSG00000157456 | -/CDC20/ESPL1/E2F1/CCNA2/ADCY1/SLC2A1/-/TERT/CCNB2 | hsa:1870/hsa:991/hsa:9700/hsa:1869/hsa:890/hsa:107/hsa:6513/hsa:1388/hsa:7015/hsa:9133 | 10 |
| hsa05206 | MicroRNAs in cancer | 8\|135 | 185\|9228 | 0.0057070 | 0.1884125 | ENSG00000026025/novel.507/ENSG00000101412/ENSG00000171791/ENSG00000146670/ENSG00000164045/ENSG00000156103/ENSG00000088305 | VIM/-/E2F1/BCL2/CDCA5/CDC25A/MMP16/DNMT3B | hsa:7431/hsa:1870/hsa:1869/hsa:596/hsa:113130/hsa:993/hsa:4325/hsa:1789 | 8 |
| hsa03410 | Base excision repair | 4\|135 | 51\|9228 | 0.0064336 | 0.1884125 | ENSG00000168496/ENSG00000065057/ENSG00000062822/ENSG00000106628 | FEN1/NTHL1/POLD1/POLD2 | hsa:2237/hsa:4913/hsa:5424/hsa:5425 | 4 |
| hsa04115 | p53 signaling pathway | 5\|135 | 89\|9228 | 0.0096848 | 0.2481735 | ENSG00000177519/ENSG00000171791/ENSG00000075218/ENSG00000184990/ENSG00000157456 | RPRM/BCL2/GTSE1/SIVA1/CCNB2 | hsa:56475/hsa:596/hsa:51512/hsa:10572/hsa:9133 | 5 |
| hsa01522 | Endocrine resistance | 5\|135 | 99\|9228 | 0.0148740 | 0.2962933 | ENSG00000164850/novel.507/ENSG00000101412/ENSG00000171791/ENSG00000164742 | GPER1/-/E2F1/BCL2/ADCY1 | hsa:2852/hsa:1870/hsa:1869/hsa:596/hsa:107 | 5 |
| hsa05161 | Hepatitis B | 7\|135 | 179\|9228 | 0.0159310 | 0.2962933 | novel.507/ENSG00000179388/ENSG00000089685/ENSG00000101412/ENSG00000171791/ENSG00000145386/novel.600 | -/EGR3/BIRC5/E2F1/BCL2/CCNA2/- | hsa:1870/hsa:1960/hsa:332/hsa:1869/hsa:596/hsa:890/hsa:1388 | 7 |
| hsa04915 | Estrogen signaling pathway | 7\|135 | 180\|9228 | 0.0163794 | 0.2962933 | ENSG00000082175/ENSG00000164850/ENSG00000160182/ENSG00000131759/ENSG00000171791/ENSG00000164742/novel.600 | PGR/GPER1/TFF1/RARA/BCL2/ADCY1/- | hsa:5241/hsa:2852/hsa:7031/hsa:5914/hsa:596/hsa:107/hsa:1388 | 7 |
| hsa00240 | Pyrimidine metabolism | 4\|135 | 68\|9228 | 0.0173440 | 0.2962933 | ENSG00000167900/ENSG00000128951/ENSG00000176890/ENSG00000168393 | TK1/DUT/TYMS/DTYMK | hsa:7083/hsa:1854/hsa:7298/hsa:1841 | 4 |
| hsa03430 | Mismatch repair | 3\|135 | 39\|9228 | 0.0190666 | 0.3006656 | ENSG00000049541/ENSG00000062822/ENSG00000106628 | RFC2/POLD1/POLD2 | hsa:5982/hsa:5424/hsa:5425 | 3 |
| hsa04928 | Parathyroid hormone synthesis, secretion and action | 5\|135 | 113\|9228 | 0.0248636 | 0.3640744 | ENSG00000171791/ENSG00000164742/novel.600/ENSG00000109062/ENSG00000156103 | BCL2/ADCY1/-/SLC9A3R1/MMP16 | hsa:596/hsa:107/hsa:1388/hsa:9368/hsa:4325 | 5 |
| hsa04911 | Insulin secretion | 4\|135 | 78\|9228 | 0.0271918 | 0.3716213 | ENSG00000104783/ENSG00000164742/ENSG00000117394/novel.600 | KCNN4/ADCY1/SLC2A1/- | hsa:3783/hsa:107/hsa:6513/hsa:1388 | 4 |
| hsa04218 | Cellular senescence | 7\|135 | 204\|9228 | 0.0300007 | 0.3739179 | novel.507/ENSG00000111206/ENSG00000101057/ENSG00000101412/ENSG00000145386/ENSG00000164045/ENSG00000157456 | -/FOXM1/MYBL2/E2F1/CCNA2/CDC25A/CCNB2 | hsa:1870/hsa:2305/hsa:4605/hsa:1869/hsa:890/hsa:993/hsa:9133 | 7 |
| hsa05222 | Small cell lung cancer | 5\|135 | 121\|9228 | 0.0321408 | 0.3739179 | novel.507/ENSG00000005961/ENSG00000101412/ENSG00000171791/ENSG00000173207 | -/ITGA2B/E2F1/BCL2/CKS1B | hsa:1870/hsa:3674/hsa:1869/hsa:596/hsa:1163 | 5 |
| hsa03440 | Homologous recombination | 3\|135 | 48\|9228 | 0.0328318 | 0.3739179 | ENSG00000154920/ENSG00000062822/ENSG00000106628 | EME1/POLD1/POLD2 | hsa:146956/hsa:5424/hsa:5425 | 3 |
| hsa04974 | Protein digestion and absorption | 4\|135 | 85\|9228 | 0.0357292 | 0.3854995 | ENSG00000092068/ENSG00000164626/ENSG00000104783/ENSG00000168542 | SLC7A8/KCNK5/KCNN4/COL3A1 | hsa:23428/hsa:8645/hsa:3783/hsa:1281 | 4 |
| hsa04080 | Neuroactive ligand-receptor interaction | 7\|135 | 219\|9228 | 0.0415858 | 0.4262548 | ENSG00000164128/ENSG00000187730/ENSG00000109255/ENSG00000168539/ENSG00000069482/ENSG00000171631/ENSG00000180739 | NPY1R/GABRD/NMU/CHRM1/GAL/P2RY6/S1PR5 | hsa:4886/hsa:2563/hsa:10874/hsa:1128/hsa:51083/hsa:5031/hsa:53637 | 7 |
| hsa03420 | Nucleotide excision repair | 3\|135 | 56\|9228 | 0.0484070 | 0.4725446 | ENSG00000049541/ENSG00000062822/ENSG00000106628 | RFC2/POLD1/POLD2 | hsa:5982/hsa:5424/hsa:5425 | 3 |
| hsa00330 | Arginine and proline metabolism | 3\|135 | 58\|9228 | 0.0527753 | 0.4917700 | ENSG00000104524/ENSG00000166165/ENSG00000137124 | PYCR3/CKB/ALDH1B1 | hsa:65263/hsa:1152/hsa:219 | 3 |
| hsa05224 | Breast cancer | 5\|135 | 147\|9228 | 0.0643002 | 0.5260750 | ENSG00000082175/ENSG00000135547/novel.507/ENSG00000101412/ENSG00000037280 | PGR/HEY2/-/E2F1/FLT4 | hsa:5241/hsa:23493/hsa:1870/hsa:1869/hsa:2324 | 5 |
| hsa00270 | Cysteine and methionine metabolism | 3\|135 | 63\|9228 | 0.0644918 | 0.5260750 | ENSG00000151224/ENSG00000088305/ENSG00000092621 | MAT1A/DNMT3B/PHGDH | hsa:4143/hsa:1789/hsa:26227 | 3 |
| hsa04540 | Gap junction | 4\|135 | 104\|9228 | 0.0658884 | 0.5260750 | ENSG00000214222/ENSG00000123416/ENSG00000164742/ENSG00000104833 | TUBBP2/TUBA1B/ADCY1/TUBB4A | hsa:203068/hsa:10376/hsa:107/hsa:10382 | 4 |
| hsa00100 | Steroid biosynthesis | 2\|135 | 29\|9228 | 0.0667217 | 0.5260750 | ENSG00000167780/ENSG00000147155 | SOAT2/EBP | hsa:8435/hsa:10682 | 2 |
| hsa04725 | Cholinergic synapse | 4\|135 | 108\|9228 | 0.0735200 | 0.5582076 | ENSG00000127588/ENSG00000168539/ENSG00000171791/ENSG00000164742 | GNG13/CHRM1/BCL2/ADCY1 | hsa:51764/hsa:1128/hsa:596/hsa:107 | 4 |
| hsa05034 | Alcoholism | 6\|135 | 205\|9228 | 0.0800632 | 0.5712069 | ENSG00000127588/ENSG00000184260/ENSG00000124635/ENSG00000180176/novel.600/ENSG00000218690 | GNG13/HIST2H2AC/HIST1H2BJ/TH/-/HIST1H2APS4 | hsa:51764/hsa:8338/hsa:8970/hsa:7054/hsa:1388/hsa:8336 | 6 |
| hsa05032 | Morphine addiction | 3\|135 | 70\|9228 | 0.0827089 | 0.5712069 | ENSG00000127588/ENSG00000187730/ENSG00000164742 | GNG13/GABRD/ADCY1 | hsa:51764/hsa:2563/hsa:107 | 3 |
| hsa01523 | Antifolate resistance | 2\|135 | 33\|9228 | 0.0835913 | 0.5712069 | ENSG00000176890/ENSG00000173638 | TYMS/SLC19A1 | hsa:7298/hsa:6573 | 2 |
| hsa04727 | GABAergic synapse | 3\|135 | 78\|9228 | 0.1058983 | 0.7002954 | ENSG00000127588/ENSG00000187730/ENSG00000164742 | GNG13/GABRD/ADCY1 | hsa:51764/hsa:2563/hsa:107 | 3 |
| hsa04926 | Relaxin signaling pathway | 4\|135 | 133\|9228 | 0.1304772 | 0.8214424 | ENSG00000127588/ENSG00000164742/novel.600/ENSG00000168542 | GNG13/ADCY1/-/COL3A1 | hsa:51764/hsa:107/hsa:1388/hsa:1281 | 4 |
| hsa00430 | Taurine and hypotaurine metabolism | 1\|135 | 10\|9228 | 0.1370918 | 0.8214424 | ENSG00000167741 | GGT6 | hsa:124975 | 1 |
| hsa00920 | Sulfur metabolism | 1\|135 | 10\|9228 | 0.1370918 | 0.8214424 | ENSG00000143416 | SELENBP1 | hsa:8991 | 1 |
| hsa04742 | Taste transduction | 2\|135 | 45\|9228 | 0.1402853 | 0.8214424 | ENSG00000127588/ENSG00000164626 | GNG13/KCNK5 | hsa:51764/hsa:8645 | 2 |
| hsa04215 | Apoptosis - multiple species | 2\|135 | 46\|9228 | 0.1453244 | 0.8214424 | ENSG00000089685/ENSG00000171791 | BIRC5/BCL2 | hsa:332/hsa:596 | 2 |
| hsa05030 | Cocaine addiction | 2\|135 | 47\|9228 | 0.1504009 | 0.8214424 | ENSG00000180176/novel.600 | TH/- | hsa:7054/hsa:1388 | 2 |
| hsa05219 | Bladder cancer | 2\|135 | 48\|9228 | 0.1555126 | 0.8214424 | novel.507/ENSG00000101412 | -/E2F1 | hsa:1870/hsa:1869 | 2 |
| hsa05163 | Human cytomegalovirus infection | 6\|135 | 251\|9228 | 0.1618925 | 0.8214424 | ENSG00000107562/novel.507/ENSG00000127588/ENSG00000101412/ENSG00000164742/novel.600 | CXCL12/-/GNG13/E2F1/ADCY1/- | hsa:6387/hsa:1870/hsa:51764/hsa:1869/hsa:107/hsa:1388 | 6 |
| hsa05226 | Gastric cancer | 4\|135 | 148\|9228 | 0.1712908 | 0.8214424 | novel.507/ENSG00000101412/ENSG00000171791/ENSG00000164362 | -/E2F1/BCL2/TERT | hsa:1870/hsa:1869/hsa:596/hsa:7015 | 4 |
| hsa00600 | Sphingolipid metabolism | 2\|135 | 52\|9228 | 0.1762705 | 0.8214424 | ENSG00000168350/ENSG00000102393 | DEGS2/GLA | hsa:123099/hsa:2717 | 2 |
| hsa04360 | Axon guidance | 5\|135 | 204\|9228 | 0.1789168 | 0.8214424 | ENSG00000107562/ENSG00000157851/ENSG00000167680/ENSG00000065320/ENSG00000044524 | CXCL12/DPYSL5/SEMA6B/NTN1/EPHA3 | hsa:6387/hsa:56896/hsa:10501/hsa:9423/hsa:2042 | 5 |
| hsa04151 | PI3K-Akt signaling pathway | 8\|135 | 376\|9228 | 0.1852787 | 0.8214424 | ENSG00000005961/ENSG00000160867/ENSG00000127588/ENSG00000168539/ENSG00000171791/novel.600/ENSG00000037280/ENSG00000118513 | ITGA2B/FGFR4/GNG13/CHRM1/BCL2/-/FLT4/MYB | hsa:3674/hsa:2264/hsa:51764/hsa:1128/hsa:596/hsa:1388/hsa:2324/hsa:4602 | 8 |
| hsa04950 | Maturity onset diabetes of the young | 1\|135 | 14\|9228 | 0.1865501 | 0.8214424 | ENSG00000116833 | NR5A2 | hsa:2494 | 1 |
| hsa00260 | Glycine, serine and threonine metabolism | 2\|135 | 54\|9228 | 0.1868088 | 0.8214424 | ENSG00000123453/ENSG00000092621 | SARDH/PHGDH | hsa:1757/hsa:26227 | 2 |
| hsa04923 | Regulation of lipolysis in adipocytes | 2\|135 | 55\|9228 | 0.1921113 | 0.8214424 | ENSG00000164128/ENSG00000164742 | NPY1R/ADCY1 | hsa:4886/hsa:107 | 2 |
| hsa00603 | Glycosphingolipid biosynthesis - globo and isoglobo series | 1\|135 | 16\|9228 | 0.2102134 | 0.8214424 | ENSG00000102393 | GLA | hsa:2717 | 1 |
| hsa00604 | Glycosphingolipid biosynthesis - ganglio series | 1\|135 | 17\|9228 | 0.2217876 | 0.8214424 | ENSG00000135454 | B4GALNT1 | hsa:2583 | 1 |
| hsa03450 | Non-homologous end-joining | 1\|135 | 17\|9228 | 0.2217876 | 0.8214424 | ENSG00000168496 | FEN1 | hsa:2237 | 1 |
| hsa04934 | Cushing syndrome | 4\|135 | 166\|9228 | 0.2252196 | 0.8214424 | novel.507/ENSG00000101412/ENSG00000164742/novel.600 | -/E2F1/ADCY1/- | hsa:1870/hsa:1869/hsa:107/hsa:1388 | 4 |
| hsa00480 | Glutathione metabolism | 2\|135 | 62\|9228 | 0.2296935 | 0.8214424 | ENSG00000167741/ENSG00000166825 | GGT6/ANPEP | hsa:124975/hsa:290 | 2 |
| hsa05033 | Nicotine addiction | 1\|135 | 18\|9228 | 0.2331934 | 0.8214424 | ENSG00000187730 | GABRD | hsa:2563 | 1 |
| hsa05215 | Prostate cancer | 3\|135 | 115\|9228 | 0.2369521 | 0.8214424 | novel.507/ENSG00000101412/ENSG00000171791 | -/E2F1/BCL2 | hsa:1870/hsa:1869/hsa:596 | 3 |
| hsa04614 | Renin-angiotensin system | 1\|135 | 19\|9228 | 0.2444332 | 0.8214424 | ENSG00000166825 | ANPEP | hsa:290 | 1 |
| hsa04927 | Cortisol synthesis and secretion | 2\|135 | 65\|9228 | 0.2459640 | 0.8214424 | ENSG00000164742/novel.600 | ADCY1/- | hsa:107/hsa:1388 | 2 |
| hsa05031 | Amphetamine addiction | 2\|135 | 65\|9228 | 0.2459640 | 0.8214424 | ENSG00000180176/novel.600 | TH/- | hsa:7054/hsa:1388 | 2 |
| hsa05169 | Epstein-Barr virus infection | 5\|135 | 230\|9228 | 0.2468771 | 0.8214424 | ENSG00000026025/novel.507/ENSG00000101412/ENSG00000171791/ENSG00000145386 | VIM/-/E2F1/BCL2/CCNA2 | hsa:7431/hsa:1870/hsa:1869/hsa:596/hsa:890 | 5 |
| hsa01230 | Biosynthesis of amino acids | 3\|135 | 118\|9228 | 0.2486835 | 0.8214424 | ENSG00000104524/ENSG00000151224/ENSG00000092621 | PYCR3/MAT1A/PHGDH | hsa:65263/hsa:4143/hsa:26227 | 3 |
| hsa00910 | Nitrogen metabolism | 1\|135 | 20\|9228 | 0.2555095 | 0.8214424 | ENSG00000074410 | CA12 | hsa:771 | 1 |
| hsa00561 | Glycerolipid metabolism | 2\|135 | 67\|9228 | 0.2568372 | 0.8214424 | ENSG00000137124/ENSG00000102393 | ALDH1B1/GLA | hsa:219/hsa:2717 | 2 |
| hsa04640 | Hematopoietic cell lineage | 2\|135 | 67\|9228 | 0.2568372 | 0.8214424 | ENSG00000005961/ENSG00000166825 | ITGA2B/ANPEP | hsa:3674/hsa:290 | 2 |
| hsa04929 | GnRH secretion | 2\|135 | 68\|9228 | 0.2622779 | 0.8214424 | ENSG00000164850/ENSG00000104783 | GPER1/KCNN4 | hsa:2852/hsa:3783 | 2 |
| hsa00053 | Ascorbate and aldarate metabolism | 1\|135 | 21\|9228 | 0.2664246 | 0.8214424 | ENSG00000137124 | ALDH1B1 | hsa:219 | 1 |
| hsa04977 | Vitamin digestion and absorption | 1\|135 | 21\|9228 | 0.2664246 | 0.8214424 | ENSG00000173638 | SLC19A1 | hsa:6573 | 1 |
| hsa05322 | Systemic lupus erythematosus | 3\|135 | 123\|9228 | 0.2684248 | 0.8214424 | ENSG00000184260/ENSG00000124635/ENSG00000218690 | HIST2H2AC/HIST1H2BJ/HIST1H2APS4 | hsa:8338/hsa:8970/hsa:8336 | 3 |
| hsa05218 | Melanoma | 2\|135 | 70\|9228 | 0.2731610 | 0.8214424 | novel.507/ENSG00000101412 | -/E2F1 | hsa:1870/hsa:1869 | 2 |
| hsa04015 | Rap1 signaling pathway | 5\|135 | 240\|9228 | 0.2746737 | 0.8214424 | ENSG00000005961/ENSG00000160867/ENSG00000164742/ENSG00000037280/ENSG00000233328 | ITGA2B/FGFR4/ADCY1/FLT4/PFN1P1 | hsa:3674/hsa:2264/hsa:107/hsa:2324/hsa:5216 | 5 |
| hsa05221 | Acute myeloid leukemia | 2\|135 | 72\|9228 | 0.2840389 | 0.8214424 | ENSG00000131759/ENSG00000145386 | RARA/CCNA2 | hsa:5914/hsa:890 | 2 |
| hsa04918 | Thyroid hormone synthesis | 2\|135 | 73\|9228 | 0.2894732 | 0.8214424 | ENSG00000164742/novel.600 | ADCY1/- | hsa:107/hsa:1388 | 2 |
| hsa04976 | Bile secretion | 2\|135 | 73\|9228 | 0.2894732 | 0.8214424 | ENSG00000164742/ENSG00000117394 | ADCY1/SLC2A1 | hsa:107/hsa:6513 | 2 |
| hsa03460 | Fanconi anemia pathway | 2\|135 | 74\|9228 | 0.2949028 | 0.8214424 | ENSG00000154920/ENSG00000221829 | EME1/FANCG | hsa:146956/hsa:2189 | 2 |
| hsa04071 | Sphingolipid signaling pathway | 3\|135 | 132\|9228 | 0.3043642 | 0.8214424 | ENSG00000168350/ENSG00000171791/ENSG00000180739 | DEGS2/BCL2/S1PR5 | hsa:123099/hsa:596/hsa:53637 | 3 |
| hsa04728 | Dopaminergic synapse | 3\|135 | 133\|9228 | 0.3083770 | 0.8214424 | ENSG00000127588/ENSG00000180176/novel.600 | GNG13/TH/- | hsa:51764/hsa:7054/hsa:1388 | 3 |
| hsa00340 | Histidine metabolism | 1\|135 | 25\|9228 | 0.3085192 | 0.8214424 | ENSG00000137124 | ALDH1B1 | hsa:219 | 1 |
| hsa04145 | Phagosome | 4\|135 | 192\|9228 | 0.3092155 | 0.8214424 | ENSG00000214222/ENSG00000123416/ENSG00000102879/ENSG00000104833 | TUBBP2/TUBA1B/CORO1A/TUBB4A | hsa:203068/hsa:10376/hsa:11151/hsa:10382 | 4 |
| hsa04917 | Prolactin signaling pathway | 2\|135 | 77\|9228 | 0.3111544 | 0.8214424 | ENSG00000114737/ENSG00000180176 | CISH/TH | hsa:1154/hsa:7054 | 2 |
| hsa05223 | Non-small cell lung cancer | 2\|135 | 77\|9228 | 0.3111544 | 0.8214424 | novel.507/ENSG00000101412 | -/E2F1 | hsa:1870/hsa:1869 | 2 |
| hsa05165 | Human papillomavirus infection | 7\|135 | 377\|9228 | 0.3141090 | 0.8214424 | ENSG00000135547/ENSG00000005961/ENSG00000101412/ENSG00000145386/ENSG00000131462/ENSG00000109062/ENSG00000164362 | HEY2/ITGA2B/E2F1/CCNA2/TUBG1/SLC9A3R1/TERT | hsa:23493/hsa:3674/hsa:1869/hsa:890/hsa:7283/hsa:9368/hsa:7015 | 7 |
| hsa04970 | Salivary secretion | 2\|135 | 78\|9228 | 0.3165558 | 0.8214424 | ENSG00000104783/ENSG00000164742 | KCNN4/ADCY1 | hsa:3783/hsa:107 | 2 |
| hsa04810 | Regulation of actin cytoskeleton | 5\|135 | 257\|9228 | 0.3232735 | 0.8283884 | ENSG00000107562/ENSG00000005961/ENSG00000160867/ENSG00000168539/ENSG00000233328 | CXCL12/ITGA2B/FGFR4/CHRM1/PFN1P1 | hsa:6387/hsa:3674/hsa:2264/hsa:1128/hsa:5216 | 5 |
| hsa00670 | One carbon pool by folate | 1\|135 | 28\|9228 | 0.3385085 | 0.8323622 | ENSG00000176890 | TYMS | hsa:7298 | 1 |
| hsa00790 | Folate biosynthesis | 1\|135 | 28\|9228 | 0.3385085 | 0.8323622 | ENSG00000180176 | TH | hsa:7054 | 1 |
| hsa04611 | Platelet activation | 3\|135 | 141\|9228 | 0.3405180 | 0.8323622 | ENSG00000005961/ENSG00000164742/ENSG00000168542 | ITGA2B/ADCY1/COL3A1 | hsa:3674/hsa:107/hsa:1281 | 3 |
| hsa00052 | Galactose metabolism | 1\|135 | 29\|9228 | 0.3482151 | 0.8323622 | ENSG00000102393 | GLA | hsa:2717 | 1 |
| hsa04672 | Intestinal immune network for IgA production | 1\|135 | 29\|9228 | 0.3482151 | 0.8323622 | ENSG00000107562 | CXCL12 | hsa:6387 | 1 |
| hsa00310 | Lysine degradation | 2\|135 | 85\|9228 | 0.3540562 | 0.8323622 | ENSG00000101945/ENSG00000137124 | SUV39H1/ALDH1B1 | hsa:6839/hsa:219 | 2 |
| hsa05214 | Glioma | 2\|135 | 87\|9228 | 0.3646472 | 0.8323622 | novel.507/ENSG00000101412 | -/E2F1 | hsa:1870/hsa:1869 | 2 |
| hsa04630 | JAK-STAT signaling pathway | 3\|135 | 148\|9228 | 0.3685770 | 0.8323622 | ENSG00000114737/ENSG00000171791/ENSG00000104998 | CISH/BCL2/IL27RA | hsa:1154/hsa:596/hsa:9466 | 3 |
| hsa01524 | Platinum drug resistance | 2\|135 | 88\|9228 | 0.3699185 | 0.8323622 | ENSG00000089685/ENSG00000171791 | BIRC5/BCL2 | hsa:332/hsa:596 | 2 |
| hsa05203 | Viral carcinogenesis | 5\|135 | 275\|9228 | 0.3757131 | 0.8323622 | ENSG00000179388/ENSG00000117399/ENSG00000124635/ENSG00000145386/novel.600 | EGR3/CDC20/HIST1H2BJ/CCNA2/- | hsa:1960/hsa:991/hsa:8970/hsa:890/hsa:1388 | 5 |
| hsa00983 | Drug metabolism - other enzymes | 2\|135 | 91\|9228 | 0.3856280 | 0.8323622 | ENSG00000167900/ENSG00000128951 | TK1/DUT | hsa:7083/hsa:1854 | 2 |
| hsa04659 | Th17 cell differentiation | 2\|135 | 91\|9228 | 0.3856280 | 0.8323622 | ENSG00000131759/ENSG00000104998 | RARA/IL27RA | hsa:5914/hsa:9466 | 2 |
| hsa01040 | Biosynthesis of unsaturated fatty acids | 1\|135 | 33\|9228 | 0.3856481 | 0.8323622 | ENSG00000097021 | ACOT7 | hsa:11332 | 1 |
| hsa00410 | beta-Alanine metabolism | 1\|135 | 34\|9228 | 0.3946680 | 0.8323622 | ENSG00000137124 | ALDH1B1 | hsa:219 | 1 |
| hsa04261 | Adrenergic signaling in cardiomyocytes | 3\|135 | 155\|9228 | 0.3964339 | 0.8323622 | ENSG00000171791/ENSG00000164742/novel.600 | BCL2/ADCY1/- | hsa:596/hsa:107/hsa:1388 | 3 |
| hsa04713 | Circadian entrainment | 2\|135 | 94\|9228 | 0.4011682 | 0.8323622 | ENSG00000127588/ENSG00000164742 | GNG13/ADCY1 | hsa:51764/hsa:107 | 2 |
| hsa05146 | Amoebiasis | 2\|135 | 94\|9228 | 0.4011682 | 0.8323622 | ENSG00000164742/ENSG00000168542 | ADCY1/COL3A1 | hsa:107/hsa:1281 | 2 |
| hsa05212 | Pancreatic cancer | 2\|135 | 96\|9228 | 0.4114269 | 0.8323622 | novel.507/ENSG00000101412 | -/E2F1 | hsa:1870/hsa:1869 | 2 |
| hsa00062 | Fatty acid elongation | 1\|135 | 36\|9228 | 0.4123152 | 0.8323622 | ENSG00000097021 | ACOT7 | hsa:11332 | 1 |
| hsa03020 | RNA polymerase | 1\|135 | 36\|9228 | 0.4123152 | 0.8323622 | ENSG00000105258 | POLR2I | hsa:5438 | 1 |
| hsa04120 | Ubiquitin mediated proteolysis | 4\|135 | 224\|9228 | 0.4157350 | 0.8323622 | ENSG00000224126/ENSG00000117399/ENSG00000108106/ENSG00000224080 | UBE2SP2/CDC20/UBE2S/UBE2FP1 | hsa:27338/hsa:991/hsa:27338/hsa:140739 | 4 |
| hsa04064 | NF-kappa B signaling pathway | 2\|135 | 98\|9228 | 0.4215998 | 0.8323622 | ENSG00000107562/ENSG00000171791 | CXCL12/BCL2 | hsa:6387/hsa:596 | 2 |
| hsa05230 | Central carbon metabolism in cancer | 2\|135 | 98\|9228 | 0.4215998 | 0.8323622 | ENSG00000141526/ENSG00000117394 | SLC16A3/SLC2A1 | hsa:9123/hsa:6513 | 2 |
| hsa05220 | Chronic myeloid leukemia | 2\|135 | 100\|9228 | 0.4316833 | 0.8323622 | novel.507/ENSG00000101412 | -/E2F1 | hsa:1870/hsa:1869 | 2 |
| hsa00051 | Fructose and mannose metabolism | 1\|135 | 39\|9228 | 0.4378329 | 0.8323622 | ENSG00000138030 | KHK | hsa:3795 | 1 |
| hsa00350 | Tyrosine metabolism | 1\|135 | 39\|9228 | 0.4378329 | 0.8323622 | ENSG00000180176 | TH | hsa:7054 | 1 |
| hsa04723 | Retrograde endocannabinoid signaling | 3\|135 | 166\|9228 | 0.4395161 | 0.8323622 | ENSG00000127588/ENSG00000187730/ENSG00000164742 | GNG13/GABRD/ADCY1 | hsa:51764/hsa:2563/hsa:107 | 3 |
| hsa04933 | AGE-RAGE signaling pathway in diabetic complications | 2\|135 | 102\|9228 | 0.4416739 | 0.8323622 | ENSG00000171791/ENSG00000168542 | BCL2/COL3A1 | hsa:596/hsa:1281 | 2 |
| hsa04211 | Longevity regulating pathway | 2\|135 | 103\|9228 | 0.4466334 | 0.8323622 | ENSG00000164742/novel.600 | ADCY1/- | hsa:107/hsa:1388 | 2 |
| hsa04925 | Aldosterone synthesis and secretion | 2\|135 | 103\|9228 | 0.4466334 | 0.8323622 | ENSG00000164742/novel.600 | ADCY1/- | hsa:107/hsa:1388 | 2 |
| hsa04724 | Glutamatergic synapse | 2\|135 | 106\|9228 | 0.4613642 | 0.8520690 | ENSG00000127588/ENSG00000164742 | GNG13/ADCY1 | hsa:51764/hsa:107 | 2 |
| hsa05210 | Colorectal cancer | 2\|135 | 108\|9228 | 0.4710584 | 0.8597247 | ENSG00000089685/ENSG00000171791 | BIRC5/BCL2 | hsa:332/hsa:596 | 2 |
| hsa04062 | Chemokine signaling pathway | 3\|135 | 175\|9228 | 0.4738970 | 0.8597247 | ENSG00000107562/ENSG00000127588/ENSG00000164742 | CXCL12/GNG13/ADCY1 | hsa:6387/hsa:51764/hsa:107 | 3 |
| hsa05152 | Tuberculosis | 3\|135 | 178\|9228 | 0.4851489 | 0.8641137 | ENSG00000171791/ENSG00000102879/ENSG00000187796 | BCL2/CORO1A/CARD9 | hsa:596/hsa:11151/hsa:64170 | 3 |
| hsa04625 | C-type lectin receptor signaling pathway | 2\|135 | 111\|9228 | 0.4854042 | 0.8641137 | ENSG00000179388/ENSG00000187796 | EGR3/CARD9 | hsa:1960/hsa:64170 | 2 |
| hsa05414 | Dilated cardiomyopathy (DCM) | 2\|135 | 112\|9228 | 0.4901329 | 0.8641137 | ENSG00000005961/ENSG00000164742 | ITGA2B/ADCY1 | hsa:3674/hsa:107 | 2 |
| hsa00620 | Pyruvate metabolism | 1\|135 | 46\|9228 | 0.4931771 | 0.8641137 | ENSG00000137124 | ALDH1B1 | hsa:219 | 1 |
| hsa04979 | Cholesterol metabolism | 1\|135 | 47\|9228 | 0.5006287 | 0.8697363 | ENSG00000167780 | SOAT2 | hsa:8435 | 1 |
| hsa04210 | Apoptosis | 3\|135 | 185\|9228 | 0.5109513 | 0.8801458 | ENSG00000123416/ENSG00000089685/ENSG00000171791 | TUBA1B/BIRC5/BCL2 | hsa:10376/hsa:332/hsa:596 | 3 |
| hsa00071 | Fatty acid degradation | 1\|135 | 49\|9228 | 0.5152073 | 0.8801458 | ENSG00000137124 | ALDH1B1 | hsa:219 | 1 |
| hsa02010 | ABC transporters | 1\|135 | 51\|9228 | 0.5293634 | 0.8895040 | ENSG00000144452 | ABCA12 | hsa:26154 | 1 |
| hsa04913 | Ovarian steroidogenesis | 1\|135 | 51\|9228 | 0.5293634 | 0.8895040 | ENSG00000164742 | ADCY1 | hsa:107 | 1 |
| hsa04935 | Growth hormone synthesis, secretion and action | 2\|135 | 122\|9228 | 0.5359011 | 0.8931686 | ENSG00000164742/novel.600 | ADCY1/- | hsa:107/hsa:1388 | 2 |
| hsa00380 | Tryptophan metabolism | 1\|135 | 54\|9228 | 0.5498317 | 0.9089960 | ENSG00000137124 | ALDH1B1 | hsa:219 | 1 |
| hsa04530 | Tight junction | 3\|135 | 200\|9228 | 0.5638766 | 0.9203946 | ENSG00000123416/ENSG00000166025/ENSG00000109062 | TUBA1B/AMOTL1/SLC9A3R1 | hsa:10376/hsa:154810/hsa:9368 | 3 |
| hsa04340 | Hedgehog signaling pathway | 1\|135 | 58\|9228 | 0.5757545 | 0.9203946 | ENSG00000171791 | BCL2 | hsa:596 | 1 |
| hsa05130 | Pathogenic Escherichia coli infection | 4\|135 | 276\|9228 | 0.5783750 | 0.9203946 | ENSG00000214222/ENSG00000123416/ENSG00000109062/ENSG00000104833 | TUBBP2/TUBA1B/SLC9A3R1/TUBB4A | hsa:203068/hsa:10376/hsa:9368/hsa:10382 | 4 |
| hsa05225 | Hepatocellular carcinoma | 3\|135 | 205\|9228 | 0.5807400 | 0.9203946 | novel.507/ENSG00000101412/ENSG00000164362 | -/E2F1/TERT | hsa:1870/hsa:1869/hsa:7015 | 3 |
| hsa00280 | Valine, leucine and isoleucine degradation | 1\|135 | 59\|9228 | 0.5820002 | 0.9203946 | ENSG00000137124 | ALDH1B1 | hsa:219 | 1 |
| hsa04022 | cGMP-PKG signaling pathway | 3\|135 | 207\|9228 | 0.5873710 | 0.9203946 | ENSG00000164742/novel.600/ENSG00000121361 | ADCY1/-/KCNJ8 | hsa:107/hsa:1388/hsa:3764 | 3 |
| hsa04330 | Notch signaling pathway | 1\|135 | 60\|9228 | 0.5881546 | 0.9203946 | ENSG00000135547 | HEY2 | hsa:23493 | 1 |
| hsa04061 | Viral protein interaction with cytokine and cytokine receptor | 1\|135 | 61\|9228 | 0.5942191 | 0.9228403 | ENSG00000107562 | CXCL12 | hsa:6387 | 1 |
| hsa05014 | Amyotrophic lateral sclerosis (ALS) | 1\|135 | 62\|9228 | 0.6001949 | 0.9251125 | ENSG00000171791 | BCL2 | hsa:596 | 1 |
| hsa04024 | cAMP signaling pathway | 3\|135 | 214\|9228 | 0.6100527 | 0.9332895 | ENSG00000164128/ENSG00000168539/ENSG00000164742 | NPY1R/CHRM1/ADCY1 | hsa:4886/hsa:1128/hsa:107 | 3 |
| hsa04550 | Signaling pathways regulating pluripotency of stem cells | 2\|135 | 144\|9228 | 0.6263926 | 0.9400776 | ENSG00000160867/ENSG00000130829 | FGFR4/DUSP9 | hsa:2264/hsa:1852 | 2 |
| hsa05167 | Kaposi sarcoma-associated herpesvirus infection | 3\|135 | 220\|9228 | 0.6288300 | 0.9400776 | novel.507/ENSG00000127588/ENSG00000101412 | -/GNG13/E2F1 | hsa:1870/hsa:51764/hsa:1869 | 3 |
| hsa04722 | Neurotrophin signaling pathway | 2\|135 | 145\|9228 | 0.6301664 | 0.9400776 | ENSG00000007264/ENSG00000171791 | MATK/BCL2 | hsa:4145/hsa:596 | 2 |
| hsa04270 | Vascular smooth muscle contraction | 2\|135 | 146\|9228 | 0.6339107 | 0.9400776 | ENSG00000122679/ENSG00000164742 | RAMP3/ADCY1 | hsa:10268/hsa:107 | 2 |
| hsa04217 | Necroptosis | 3\|135 | 223\|9228 | 0.6379854 | 0.9400776 | ENSG00000184260/ENSG00000171791/ENSG00000218690 | HIST2H2AC/BCL2/HIST1H2APS4 | hsa:8338/hsa:596/hsa:8336 | 3 |
| hsa04610 | Complement and coagulation cascades | 1\|135 | 71\|9228 | 0.6501945 | 0.9400776 | ENSG00000131187 | F12 | hsa:2161 | 1 |
| hsa04920 | Adipocytokine signaling pathway | 1\|135 | 71\|9228 | 0.6501945 | 0.9400776 | ENSG00000117394 | SLC2A1 | hsa:6513 | 1 |
| hsa04720 | Long-term potentiation | 1\|135 | 72\|9228 | 0.6553516 | 0.9400776 | ENSG00000164742 | ADCY1 | hsa:107 | 1 |
| hsa04066 | HIF-1 signaling pathway | 2\|135 | 152\|9228 | 0.6557614 | 0.9400776 | ENSG00000171791/ENSG00000117394 | BCL2/SLC2A1 | hsa:596/hsa:6513 | 2 |
| hsa04978 | Mineral absorption | 1\|135 | 73\|9228 | 0.6604332 | 0.9402001 | ENSG00000198417 | MT1F | hsa:4494 | 1 |
| hsa05323 | Rheumatoid arthritis | 1\|135 | 75\|9228 | 0.6703745 | 0.9450555 | ENSG00000107562 | CXCL12 | hsa:6387 | 1 |
| hsa04213 | Longevity regulating pathway - multiple species | 1\|135 | 77\|9228 | 0.6800267 | 0.9450555 | ENSG00000164742 | ADCY1 | hsa:107 | 1 |
| hsa04371 | Apelin signaling pathway | 2\|135 | 161\|9228 | 0.6865859 | 0.9450555 | ENSG00000127588/ENSG00000164742 | GNG13/ADCY1 | hsa:51764/hsa:107 | 2 |
| hsa05170 | Human immunodeficiency virus 1 infection | 3\|135 | 242\|9228 | 0.6923113 | 0.9450555 | ENSG00000127588/ENSG00000171791/ENSG00000157456 | GNG13/BCL2/CCNB2 | hsa:51764/hsa:596/hsa:9133 | 3 |
| hsa03320 | PPAR signaling pathway | 1\|135 | 81\|9228 | 0.6984976 | 0.9450555 | ENSG00000214456 | PLIN5 | hsa:440503 | 1 |
| hsa04068 | FoxO signaling pathway | 2\|135 | 165\|9228 | 0.6995488 | 0.9450555 | ENSG00000166851/ENSG00000157456 | PLK1/CCNB2 | hsa:5347/hsa:9133 | 2 |
| hsa03013 | RNA transport | 3\|135 | 246\|9228 | 0.7029419 | 0.9450555 | novel.674/ENSG00000013810/ENSG00000183684 | -/TACC3/ALYREF | hsa:79897/hsa:10460/hsa:10189 | 3 |
| hsa04510 | Focal adhesion | 3\|135 | 249\|9228 | 0.7107318 | 0.9450555 | ENSG00000005961/ENSG00000171791/ENSG00000037280 | ITGA2B/BCL2/FLT4 | hsa:3674/hsa:596/hsa:2324 | 3 |
| hsa05160 | Hepatitis C | 2\|135 | 173\|9228 | 0.7241519 | 0.9450555 | novel.507/ENSG00000101412 | -/E2F1 | hsa:1870/hsa:1869 | 2 |
| hsa04971 | Gastric acid secretion | 1\|135 | 88\|9228 | 0.7283102 | 0.9450555 | ENSG00000164742 | ADCY1 | hsa:107 | 1 |
| hsa05211 | Renal cell carcinoma | 1\|135 | 90\|9228 | 0.7362772 | 0.9450555 | ENSG00000117394 | SLC2A1 | hsa:6513 | 1 |
| hsa04512 | ECM-receptor interaction | 1\|135 | 91\|9228 | 0.7401733 | 0.9450555 | ENSG00000005961 | ITGA2B | hsa:3674 | 1 |
| hsa04137 | Mitophagy - animal | 2\|135 | 180\|9228 | 0.7442758 | 0.9450555 | ENSG00000254337/ENSG00000101412 | AC083967.1/E2F1 | hsa:55626/hsa:1869 | 2 |
| hsa05142 | Chagas disease (American trypanosomiasis) | 1\|135 | 93\|9228 | 0.7477949 | 0.9450555 | ENSG00000164742 | ADCY1 | hsa:107 | 1 |
| hsa04972 | Pancreatic secretion | 1\|135 | 94\|9228 | 0.7515221 | 0.9450555 | ENSG00000164742 | ADCY1 | hsa:107 | 1 |
| hsa04014 | Ras signaling pathway | 3\|135 | 269\|9228 | 0.7587248 | 0.9450555 | ENSG00000160867/ENSG00000127588/ENSG00000037280 | FGFR4/GNG13/FLT4 | hsa:2264/hsa:51764/hsa:2324 | 3 |
| hsa04750 | Inflammatory mediator regulation of TRP channels | 1\|135 | 96\|9228 | 0.7588132 | 0.9450555 | ENSG00000164742 | ADCY1 | hsa:107 | 1 |
| hsa01521 | EGFR tyrosine kinase inhibitor resistance | 1\|135 | 97\|9228 | 0.7623787 | 0.9450555 | ENSG00000171791 | BCL2 | hsa:596 | 1 |
| hsa05418 | Fluid shear stress and atherosclerosis | 2\|135 | 188\|9228 | 0.7657349 | 0.9450555 | ENSG00000005961/ENSG00000171791 | ITGA2B/BCL2 | hsa:3674/hsa:596 | 2 |
| hsa04916 | Melanogenesis | 1\|135 | 102\|9228 | 0.7794364 | 0.9450555 | ENSG00000164742 | ADCY1 | hsa:107 | 1 |
| hsa04726 | Serotonergic synapse | 1\|135 | 103\|9228 | 0.7826992 | 0.9450555 | ENSG00000127588 | GNG13 | hsa:51764 | 1 |
| hsa04621 | NOD-like receptor signaling pathway | 2\|135 | 195\|9228 | 0.7832226 | 0.9450555 | ENSG00000171791/ENSG00000187796 | BCL2/CARD9 | hsa:596/hsa:64170 | 2 |
| hsa00010 | Glycolysis / Gluconeogenesis | 1\|135 | 104\|9228 | 0.7859140 | 0.9450555 | ENSG00000137124 | ALDH1B1 | hsa:219 | 1 |
| hsa04912 | GnRH signaling pathway | 1\|135 | 104\|9228 | 0.7859140 | 0.9450555 | ENSG00000164742 | ADCY1 | hsa:107 | 1 |
| hsa03018 | RNA degradation | 1\|135 | 105\|9228 | 0.7890817 | 0.9450555 | ENSG00000130520 | LSM4 | hsa:25804 | 1 |
| hsa05410 | Hypertrophic cardiomyopathy (HCM) | 1\|135 | 106\|9228 | 0.7922028 | 0.9450555 | ENSG00000005961 | ITGA2B | hsa:3674 | 1 |
| hsa00564 | Glycerophospholipid metabolism | 1\|135 | 107\|9228 | 0.7952781 | 0.9450555 | ENSG00000143845 | ETNK2 | hsa:55224 | 1 |
| hsa05412 | Arrhythmogenic right ventricular cardiomyopathy (ARVC) | 1\|135 | 107\|9228 | 0.7952781 | 0.9450555 | ENSG00000005961 | ITGA2B | hsa:3674 | 1 |
| hsa04020 | Calcium signaling pathway | 2\|135 | 202\|9228 | 0.7995634 | 0.9450555 | ENSG00000168539/ENSG00000164742 | CHRM1/ADCY1 | hsa:1128/hsa:107 | 2 |
| hsa05202 | Transcriptional misregulation in cancer | 2\|135 | 205\|9228 | 0.8062291 | 0.9450555 | ENSG00000131759/ENSG00000145386 | RARA/CCNA2 | hsa:5914/hsa:890 | 2 |
| hsa05100 | Bacterial invasion of epithelial cells | 1\|135 | 112\|9228 | 0.8099898 | 0.9450555 | ENSG00000116670 | MAD2L2 | hsa:10459 | 1 |
| hsa04060 | Cytokine-cytokine receptor interaction | 2\|135 | 208\|9228 | 0.8126987 | 0.9450555 | ENSG00000107562/ENSG00000104998 | CXCL12/IL27RA | hsa:6387/hsa:9466 | 2 |
| hsa04141 | Protein processing in endoplasmic reticulum | 2\|135 | 210\|9228 | 0.8169048 | 0.9450555 | ENSG00000171791/novel.600 | BCL2/- | hsa:596/hsa:1388 | 2 |
| hsa04931 | Insulin resistance | 1\|135 | 116\|9228 | 0.8209995 | 0.9450555 | ENSG00000117394 | SLC2A1 | hsa:6513 | 1 |
| hsa04740 | Olfactory transduction | 1\|135 | 123\|9228 | 0.8387641 | 0.9450555 | ENSG00000127588 | GNG13 | hsa:51764 | 1 |
| hsa04922 | Glucagon signaling pathway | 1\|135 | 123\|9228 | 0.8387641 | 0.9450555 | ENSG00000117394 | SLC2A1 | hsa:6513 | 1 |
| hsa05145 | Toxoplasmosis | 1\|135 | 125\|9228 | 0.8435102 | 0.9450555 | ENSG00000171791 | BCL2 | hsa:596 | 1 |
| hsa03015 | mRNA surveillance pathway | 1\|135 | 126\|9228 | 0.8458310 | 0.9450555 | ENSG00000183684 | ALYREF | hsa:10189 | 1 |
| hsa04668 | TNF signaling pathway | 1\|135 | 126\|9228 | 0.8458310 | 0.9450555 | novel.600 |  | hsa:1388 | 1 |
| hsa04514 | Cell adhesion molecules (CAMs) | 1\|135 | 128\|9228 | 0.8503706 | 0.9450555 | ENSG00000105695 | MAG | hsa:4099 | 1 |
| hsa03040 | Spliceosome | 2\|135 | 229\|9228 | 0.8528549 | 0.9450555 | ENSG00000130520/ENSG00000183684 | LSM4/ALYREF | hsa:25804/hsa:10189 | 2 |
| hsa04152 | AMPK signaling pathway | 1\|135 | 134\|9228 | 0.8632083 | 0.9513855 | ENSG00000145386 | CCNA2 | hsa:890 | 1 |
| hsa04010 | MAPK signaling pathway | 3\|135 | 331\|9228 | 0.8680857 | 0.9516448 | ENSG00000160867/ENSG00000130829/ENSG00000037280 | FGFR4/DUSP9/FLT4 | hsa:2264/hsa:1852/hsa:2324 | 3 |
| hsa05012 | Parkinson disease | 4\|135 | 430\|9228 | 0.8806956 | 0.9542349 | ENSG00000214222/ENSG00000123416/ENSG00000180176/ENSG00000104833 | TUBBP2/TUBA1B/TH/TUBB4A | hsa:203068/hsa:10376/hsa:7054/hsa:10382 | 4 |
| hsa05162 | Measles | 1\|135 | 144\|9228 | 0.8822196 | 0.9542349 | ENSG00000171791 | BCL2 | hsa:596 | 1 |
| hsa04670 | Leukocyte transendothelial migration | 1\|135 | 147\|9228 | 0.8873936 | 0.9542349 | ENSG00000107562 | CXCL12 | hsa:6387 | 1 |
| hsa04919 | Thyroid hormone signaling pathway | 1\|135 | 148\|9228 | 0.8890676 | 0.9542349 | ENSG00000117394 | SLC2A1 | hsa:6513 | 1 |
| hsa00230 | Purine metabolism | 1\|135 | 153\|9228 | 0.8970744 | 0.9578138 | ENSG00000164742 | ADCY1 | hsa:107 | 1 |
| hsa04140 | Autophagy - animal | 2\|135 | 270\|9228 | 0.9095947 | 0.9608993 | ENSG00000254337/ENSG00000171791 | AC083967.1/BCL2 | hsa:55626/hsa:596 | 2 |
| hsa04142 | Lysosome | 1\|135 | 162\|9228 | 0.9100677 | 0.9608993 | ENSG00000102393 | GLA | hsa:2717 | 1 |
| hsa04072 | Phospholipase D signaling pathway | 1\|135 | 165\|9228 | 0.9140261 | 0.9608993 | ENSG00000164742 | ADCY1 | hsa:107 | 1 |
| hsa04310 | Wnt signaling pathway | 1\|135 | 169\|9228 | 0.9190362 | 0.9612368 | ENSG00000175792 | RUVBL1 | hsa:8607 | 1 |
| hsa04921 | Oxytocin signaling pathway | 1\|135 | 180\|9228 | 0.9313685 | 0.9674295 | ENSG00000164742 | ADCY1 | hsa:107 | 1 |
| hsa01200 | Carbon metabolism | 1\|135 | 183\|9228 | 0.9343953 | 0.9674295 | ENSG00000092621 | PHGDH | hsa:26227 | 1 |
| hsa04390 | Hippo signaling pathway | 1\|135 | 193\|9228 | 0.9435591 | 0.9720082 | ENSG00000089685 | BIRC5 | hsa:332 | 1 |
| hsa05132 | Salmonella infection | 2\|135 | 317\|9228 | 0.9493804 | 0.9731149 | ENSG00000171791/ENSG00000233328 | BCL2/PFN1P1 | hsa:596/hsa:5216 | 2 |
| hsa04144 | Endocytosis | 2\|135 | 343\|9228 | 0.9635768 | 0.9806632 | ENSG00000160867/ENSG00000024422 | FGFR4/EHD2 | hsa:2264/hsa:30846 | 2 |
| hsa05205 | Proteoglycans in cancer | 1\|135 | 238\|9228 | 0.9713811 | 0.9806632 | ENSG00000090447 | TFAP4 | hsa:7023 | 1 |
| hsa05017 | Spinocerebellar ataxia | 1\|135 | 240\|9228 | 0.9722342 | 0.9806632 | ENSG00000254337 | AC083967.1 | hsa:55626 | 1 |
| hsa05131 | Shigellosis | 2\|135 | 375\|9228 | 0.9758795 | 0.9806632 | ENSG00000171791/ENSG00000233328 | BCL2/PFN1P1 | hsa:596/hsa:5216 | 2 |
| hsa04714 | Thermogenesis | 1\|135 | 360\|9228 | 0.9955384 | 0.9955384 | ENSG00000164742 | ADCY1 | hsa:107 | 1 |
